# Sex Pheromones of Two Leafminer Species, *Antispila oinophylla* and *Holocacista rivillei* (Lepidoptera: Heliozelidae) Infesting Grapevine in Italy

**DOI:** 10.1007/s10886-018-1036-z

**Published:** 2018-12-14

**Authors:** Hong-Lei Wang, Mario Baldessari, Gianfranco Anfora, Erik J. van Nieukerken, Christer Löfstedt

**Affiliations:** 1 Department of Biology, Lund University, Sölvegatan 37, 223 62 Lund, Sweden; 2 Technology Transfer Centre, Fondazione Edmund Mach, San Michele all´Adige, Italy; 3 Research and Innovation Centre, Fondazione Edmund Mach, San Michele all’Adige, Italy; 4 Centre Agriculture Food Environment, University of Trento, San Michele all’Adige, Italy; 5 Naturalis Biodiversity Center, Leiden, The Netherlands

**Keywords:** Sex attractant, *Vitis vinifera*, (*Z*)-5-dodecenal, (*Z*)-5-tetradecenal, (*Z*)-7-tetradecenal

## Abstract

Two heliozelid species, *Antispila oinophylla* van Nieukerken & Wagner and *Holocacista rivillei* (Stainton) severely infest Italian grapevines. The volatile pheromones from calling females were collected by solid phase micro extraction (SPME) and analyzed by gas chromatography with electroantennographic detection (GC-EAD). Two compounds from *A. oinophylla* females eliciting electrophysiological activity from the conspecific male antenna were identified as (*Z*)-5-tetradecenal and (*Z*)-7-tetradecenal by coupled gas chromatography/mass spectrometry (GC/MS) analysis. SPME collections from *H. rivillei* produced no GC-EAD active compounds but analysis of fatty acyl moieties in the pheromone gland, demonstrated the presence of the putative pheromone biosynthetic precursors (*Z*)-5-dodecenoic acid and (*Z*)-7-tetradecenoic acid. Field trapping experiments in Italy confirmed that (*Z*)-5-tetradecenal and (*Z*)-7-tetradecenal are essential for the attraction of male *A. oinophylla* in a blend ratio of 15:100 respectively, whereas (*Z*)-5-dodecenal and (*Z*)-7-tetradecenal attract male *H. rivillei* in a blend ratio of 100:6.

## Introduction

Several leaf mining insects from the moth family Heliozelidae infest host plants belonging to the grape family Vitaceae. They may cause severe damage to their hosts and seriously affect grape production (Duso et al. 2011, 2013; van Nieukerken and Geertsema 2015). Over the past decades, various pesticides have been used in grape production to control these and other pests, resulting in residues contaminating grapes and eventually migrating into the wine (Andrey and Amstutz 2000; Cabras et al. 1995). Due to the increasing public concern over the safety of table grapes and wine, the use of pesticides is not recommended, especially in the period around harvest when the leafminers may be highly abundant. Sex pheromones may provide alternative approaches to traditional chemical pesticides for pest control in integrated pest management programs (Pertot et al. 2017).

In Italy, larvae of *Antispila oinophylla* van Nieukerken & Wagner and *Holocacista rivillei* (Stainton) may cause serious damage in vineyards (van Nieukerken et al. 2012). Although they are considered secondary pests, in recent years severe outbreaks have taken place in some Italian grape growing areas. *Holocacista rivillei* is a native of the Mediterranean Basin and has always been associated with the grapevine in several viticultural areas of southern Europe. However, infestation rarely reaches economically-damaging levels, probably as a result of the rich natural enemy communities. Local outbreaks of *H. rivillei* are probably induced by the disruption of interactions between the pest and its antagonists due to the repeated use of non-selective pesticides. *Antispila oinophylla* is a North American grapevine leafminer invading Italian vineyards since 2006. Significant infestations are commonly encountered in vineyards in newly colonized areas such as South Tyrol.

The compositions of female-produced sex pheromones in heliozelid moths remain largely unknown. Based on a previous field screening test in Hungary, the heliozelid moth *Antispila treitschkiella* (Fischer von Röslerstamm) was recorded in traps baited with (*Z*)-7-tetradecenal (Z7–14:Ald) (Tóth et al. 1992). As a substitute for a properly identified pheromone of *A. oinophylla*, traps for the olive moth *Prays oleae* (Bernard) baited with Z7–14:Ald (Campion et al. 1979; Renou et al. 1979) were used in combination with visual inspection to monitor its flight dynamics in vineyards (Baldessari et al. 2010). Recently, we reported the sex pheromone from a newly-discovered grape-feeding leafminer, *Holocacista capensis,* infesting South African table grapes (van Nieukerken and Geertsema 2015). The pheromone was identified as a binary mixture of 14-carbon, monounsaturated aldehydes, (*Z*)-5-tetradecenal (Z5–14:Ald) and Z7–14:Ald (Wang et al. 2015).

In the present study, we investigated the female-produced sex pheromones of *A. oinophylla* and *H. rivillei.* Volatiles from calling females were collected by solid phase micro extraction (SPME) and analysed by gas chromatography coupled with electroantennographic detection (GC-EAD) and mass spectrometry (GC/MS). Due to the difficulty of obtaining sufficient amounts of pheromone from calling females of *H. rivillei* for identification, the pheromone biosynthetic precursors were also analyzed in an attempt to predict the structures of pheromone components from the composition of the precursors. Synthetic pheromone candidates were assayed by trapping experiments in the field.

## Methods and Materials

### Insects

Cocoons of *A. oinophylla* and *H. rivillei* were collected in late March (winter generation) and early July (summer generation) in 2013 from the wine grape plantations of Trentino Region, Italy, and sent to Lund where they were stored at 23 °C, 60% RH and a 17:7 h L:D cycle. Moths from the different generations started to emerge in mid-to-late May and late July, respectively. Newly emerged moths were separated into individual vials to prevent mating and then sexed based on the difference in relative length of antenna. Males have significantly longer antennae than females, as confirmed by dissection of the genitalia (unpublished results). The adults were kept individually until used in experiments.

### Chemicals

Reference compounds of different origin and purity were available for the identification work from our laboratory collection of pheromone compounds. For chemical identification and electrophysiological experiments, (*Z*)-5-dodecenal (Z5–12:Ald), (*E*)-5-dodecenal (E5–12:Ald), Z5–14:Ald, (*E*)-5-tetradecenal (E5–14:Ald), Z7–14:Ald and (*E*)-7-tetradecenal (E7–14:Ald) were prepared by oxidation of corresponding alcohols with pyridinium chlorochromate as described by Corey and Suggs (1975). The corresponding fatty acids and their methyl esters were also prepared from the respective alcohols as described in Wang et al. (2010). For field experiments, Z5–14:Ald (chemical purity 99.6%) and Z7–14:Ald (chemical purity 98.2%) were purchased from Pherobank (Wageningen, The Netherlands), and Z5–12:Ald (chemical purity 98.7%) was prepared in our laboratory from the corresponding alcohol according to Corey and Suggs (1975)

### Isolation of Sex Pheromones and Pheromone Precursors

To collect volatiles, an SPME fiber coated with PDMS (7 μm film thickness, Supelco, USA) was inserted into a 5 mL gas tight syringe (Hamilton, Switzerland) to collect the headspace volatiles from 0 to 5 d old virgin female adults contained in the syringe. Volatile samples from the female moths (up to 30 individuals for *A. oinophylla*, and up to 8 individuals for *H. rivillei*) were collected repeatedly for 24 h, 48 h or 60 h. Newly emerged females were added into the syringe chamber during the headspace collection. The SPME samples were immediately subjected to GC/MS or GC-EAD analysis.

For the pheromone precursor analysis, 5–6 female abdominal tips dissected from 1- to 2-d old virgin females were combined and extracted with 10 μL chloroform/methanol (2:1 *v*:*v*) at room temperature for 24 h. The fatty acyl contents in the lipid extracts were transformed into corresponding methyl esters by base methanolysis as described in Bjostad and Roelofs (1984), and then analyzed by GC/MS.

### Electrophysiology

The electrophysiological activities of the SPME samples and synthetic reference compounds were determined by analysis on an Agilent 7890 gas chromatograph equipped with a flame ionization detector (FID) (Agilent, Santa Clara, California) and an electroantennographic detector (EAD). An HP-INNOWax column (30 m × 0.25 mm i.d., and 0.25 μm film thickness; J&W Scientific, USA) was used in the GC, where the inlet was set at 230 °C, the transfer line was heated at 255 °C and the detector was set at 280 °C. Hydrogen was used as the carrier gas, and at the end of the column a 1:1 division of the GC effluent was directed to the FID and EAD, respectively. After cutting off the tips, the antennae associated with the head of a 1–2 d old male were mounted to a PRG-2 EAG (10x gain) probe (Syntech, Kirchzarten, Germany) using conductive gel (Blågel, Cefar, Malmö, Sweden), and charcoal-filtered and humidified air passed over the antennal preparation. The GC oven was programmed from 80 °C for 1 min, at a rate of 10 °C/min to 210 °C, held for 10 min and then to 230 °C at 10 °C/min for 10 min. In the case of SPME samples, the fiber containing absorbed volatiles was injected into the GC inlet and held for 5 min. Data were collected with the software GC-EAD Pro Version 4.1 (Syntech).

### Gas Chromatography/Mass Spectrometry

The SPME samples of volatiles collected from females and the fatty acyl methyl esters from gland extracts were analyzed on an Agilent 5975 mass-selective detector coupled to an Agilent 6890 gas chromatograph (Agilent). An HP-INNOWax column (30 m × 0.25 mm i.d., and 0.25 μm film thickness; J&W Scientific, USA) and a HP-5MS column (30 m × 0.25 mm i.d., and 0.25 μm film thickness; J&W Scientific, USA) were used for the analyses. Helium was used as carrier gas at a constant flow of 0.8 mL/min corresponding to linear velocity of 33 cm/s. The oven temperature program was the same as in the GC-EAD analyses.

For both SPME samples and gland fatty acyl extracts, compounds were identified based on comparison of their retention times and mass spectra with those of synthetic references on both polar and non-polar columns. Double bond positions in the putative fatty acyl pheromone precursors were localized by GC/MS analysis of the dimethyldisulphide (DMDS) adducts of corresponding methyl esters, prepared according to Dunkelblum et al. (1985). For the analysis of DMDS-adducts of fatty acyl precursors the HP-5MS column was used and the oven was programmed at 80 °C for 2 min, then to 140 °C at a rate of 15 °C/min, and finally to 260 °C at 5 °C/min, held for 20 min.

### Field Experiments

The first field trials were carried out from 29 July to 17 September 2013 for *A. oinophylla*, and 31 July to 23 September for *H. rivillei*, in the same vineyards where the cocoons had been collected. The *A. oinophylla* trapping experiments were carried out in wine grape plantations of the cultivars Chardonnay at Pochi di Salorno, Bolzano, Italy (470 m a.s.l., 46°14’N 11°13E). The field trials on *H. rivillei* were carried out in a vineyard with the cultivar Pinot gris at Avio, Trento, Italy (124 m a.s.l., 45°41’N 10°55′E). The vineyards received fungicide treatments but insecticides were not applied. Synthetic blends to be tested were prepared in hexane containing 0.02% of the antioxidant butylated hydroxytoluene (BHT) and loaded on the rubber septa (Catalogue no. 224100–020, Wheaton Science Products, Millville, NJ, USA) used as dispensers. For each species, treatments included a ternary mixture (A), three binary mixtures (B-D), three single components (E-G) and hexane alone as solvent control (H), as well as the commercial lures for the olive moth, *P. oleae* (I) containing Z7–14:Ald (AgriSense LLC) for comparison. The blend ratios as shown in Figs. 4 and 5 were set as found in the fatty acyl precursor composition. Except for the two control groups (H and I), the dosage of treatment A-G was set at 100 μg of the major component per bait, i.e. Z7–14 Ald for *A. oinophylla* and Z5–12:Ald for *H. rivillei*.

A second round of field trials was carried out during five weeks (W1-W5) between late May and late June 2014, to compare different dosages of the most active binary mixture for each species.

The actual blend ratios in all the treatments were confirmed by GC-FID before the field test. Five trap replicates were used for each treatment. Delta-traps with sticky inserts (Csalomon, Budapest, Hungary) were baited with the lures and suspended below the vine canopy, with the different treatments in random sequences. Traps within a replicate were randomly placed in two rows of grapevine in the orchard, with a distance of 15 m between traps and 20 m between rows. Traps were inspected once a week and redistributed within each replicate so as to minimize the potential position effect. The trapped moths were identified by their morphological characteristics.

### Statistical Analyses

Differences in trap catch between treatments were compared using one-way ANOVA analysis followed by a Least Significant Difference (LSD) test (*P* < 0.01). All analyses were performed using SPSS ver.16.

## Results

### Analyses of Volatile Collections

**Fig. 1:**
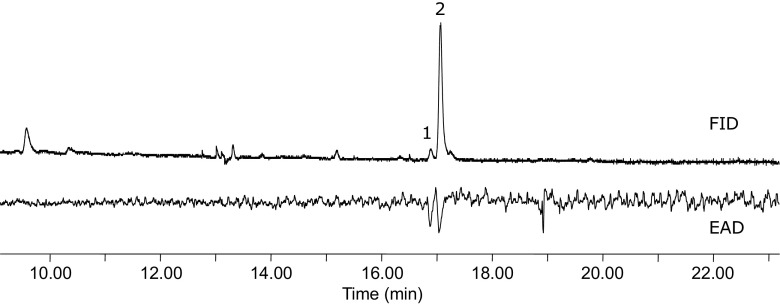
Coupled gas chromatographic-electroantennographic (GC-EAD) analysis of an SPME collection from female *Antispila oinophylla*. The upper trace is the chromatogram monitored with flame ionization detector (FID) and the lower trace is the antennal response from a conspecific male antenna. The SPME sample was collected for 24 h from approximately 30 4–5 d old, virgin female moths. Compound 1 was identified as (*Z*)-5-tetradecenal (Z5–14:Ald) and compound 2 as (Z)-7-tetradecenal (Z7–14:Ald)

The antennae of male *A. oinophylla* showed two repeatable responses to the SPME collections from conspecific female in GC-EAD analyses (Fig. 1). The active components eluted close to each other, the first component in trace amounts and the second in a larger amounts. The mass spectra of the two compounds showed the same characteristic fragments but with different ion ratios as did the two aldehydes previously identified from the South African leafminer *H. capensis*, including a molecular ion at *m*/*z* 210, a [M-18]^+^ fragment at *m/z* 192 and a [M-44]^+^fragment at *m*/*z* 166 (Wang et al. 2015). These compounds were subsequently confirmed as Z5–14:Ald and Z7–14:Ald (compounds 1 and 2 respectively in Fig. 1), by comparing the retention times and mass spectra with those of corresponding synthetic references on both the polar and nonpolar column.

However, in GC-EAD analyses of SPME collections from female *H. rivillei*, no responses were detected from conspecific male antennae.

### Analyses of Pheromone Precursors

**Fig. 2:**
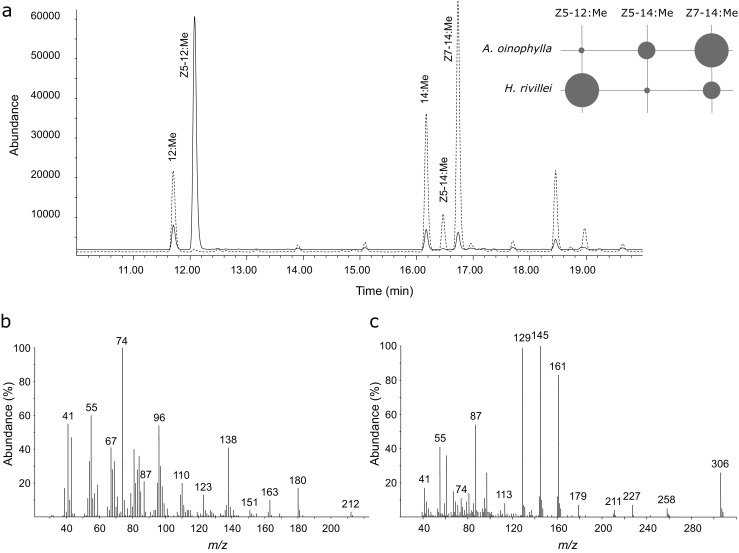
GC/MS analysis of fatty acyl pheromone precursors from pheromone glands of *Antispila oinophylla* and *Holocacista rivillei* in the form of methyl esters. **a** Total ion current (TIC) chromatogram of C_12_-C_14_ fatty acids in the gland of *Antispila oinophylla* (dashed line) and *Holocacista rivillei* (solid line). The insert diagram shows the composition of mono-unsaturated C_12_ and C_14_ acids, where the size of the circles indicates approximately their abundance. **b** Mass spectrum of the C_12_ mono-unsaturated methyl ester. **c** Mass spectrum of the DMDS-adduct of the C_12_ mono-unsaturated methyl ester

As another approach to identification of pheromone components, the total lipids of the abdominal tips, where the pheromone gland is most likely located, were extracted and subjected to methanolysis. The resulting fatty acid methyl esters were analyzed by GC/MS to investigate putative biosynthetic precursors of the pheromone components. In addition to the ubiquitous lauric acid, myristic acid, palmitic acid, palmitoleic acid, stearic acid, oleic acid, linoleic acid and linolenic acid, two unusual mono-unsaturated C_14_ acids and one mono-unsaturated C_12_ acid were found in the abdominal tips of both species, but in very different ratios (Fig. 2a). The two mono-unsaturated C_14_ acids were confirmed as identical to the previously reported methyl (*Z*)-5-tetradecenoate (Z5–14:Me) and methyl (*Z*)-7-tetradecenoate (Z7–14:Me) found in *H. capensis* (Wang et al. 2015), by comparing both methyl ester and DMDS-adduct spectra with those of the synthetic compounds. In the latter case, the double bond position at Δ5 and Δ7 were indicated by a pair of diagnostic ions at *m/z* 161/173 and 189/145, respectively, plus a molecular ion at *m*/*z* 334. The ¨Z¨ configuration of the double bond was determined by comparing the retention times with those of the synthetic geometric isomers. In *A. oinophylla*, the ratio between the two C_14_ precursors was approximately the same as the ratio between the corresponding aldehydes found in the volatile collections.

The methyl ester of the mono-unsaturated C_12_ acid was more abundant in *H. rivillei* compared to *A. oinophylla* (Fig. 2a). The mass spectrum of this compound showed a molecular ion at *m*/*z* 212, a [M-32]^+^fragment at *m*/*z* 180, a [M-74]^+^fragment at *m*/*z* 138, and a combination of the fragments at *m*/*z* 74 and 87 (Fig. 2b). The corresponding DMDS adduct showed a pair of diagnostic ions at *m/z* 161/145, and a molecular ion at *m*/*z* 306, indicating a double bond between carbon atoms 5 and 6 in the C_12_ acyl skeleton (Fig. 2c). Finally, this compound was confirmed as methyl (*Z*)-5-dodecenoate (Z5–12:Me), by comparing the mass spectrum and retention time with those of the synthetic geometric isomers on both columns.

**Fig. 3:**
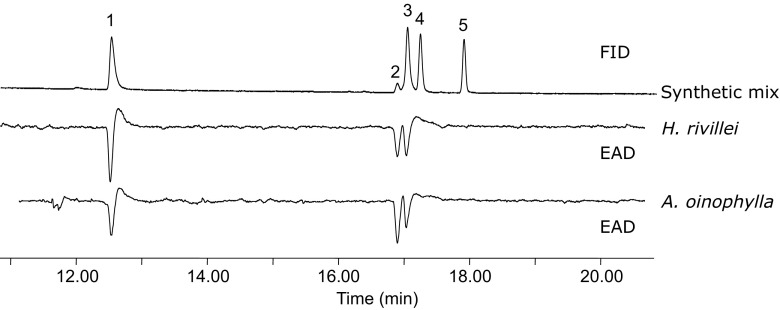
GC-EAD responses from male *Antispila oinophylla* and *Holocacista rivillei* to synthetic pheromone compounds. Compounds in the mixture: 1 = (*Z*)-5-dodecenal, 2 = (*Z*)-5-tetradecenal, 3 = (*Z*)-7-tetradecenal, 4 = dodecan-1-ol, and 5 = dodecyl acrylate (the latter two compounds were found in trace amount in the SPME sample from female *A. oinophylla*)

These results suggest that female *H. rivillei* might produce a pheromone compound with corresponding chain length and position of unsaturation, but the release rate of these compounds was below the limit of detection in our collection of volatiles. Indeed, the synthetic compounds, Z5–12:Ald, Z5–14:Ald and Z7–14:Ald elicited strong responses from male *H. rivillei*, confirming the antennal activity of these aldehydes (Fig. 3). Interestingly, antennae from male *A. oinophylla* also showed a strong response to Z5–12:Ald, although the corresponding fatty acyl precursor Z5–12:Me was found in very low amounts in this species compared to Z5–14:Me and Z7–14:Me (Fig. 2a).

### Field Tests

**Fig. 4:**
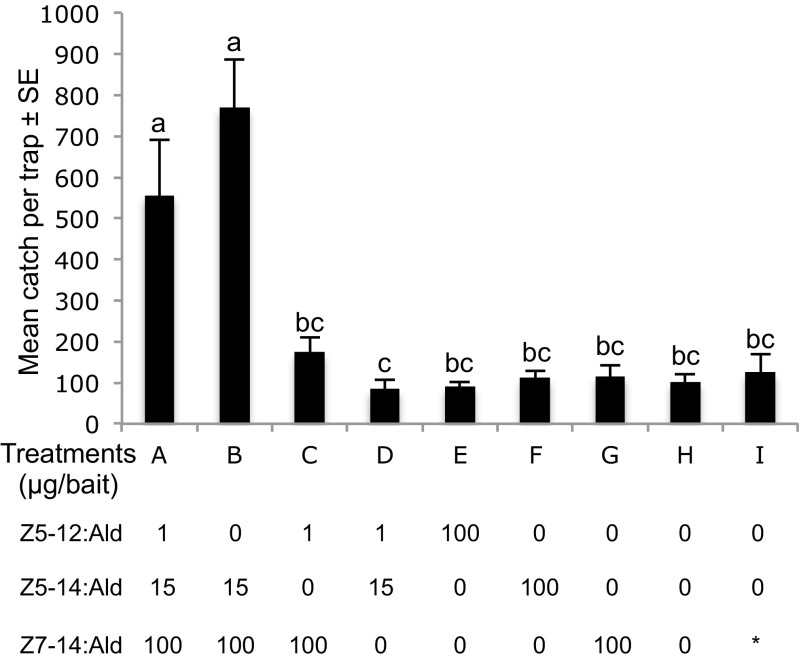
Trap catches of male *Antispila oinophylla* by different pheromone component candidates presented singly and in mixtures in a vineyard located in Pochi di Salorno, Italy (2013). Bars with the same letter are not significantly different (*P* > 0.05, one-way-ANOVA followed by a LSD test; 5 replicates for each treatment). Asterisk indicates that the treatment I is the commercial bait of the olive moth, *Prays oleae* containing a single component Z7–14:Ald at unknown concentration

**Fig. 5:**
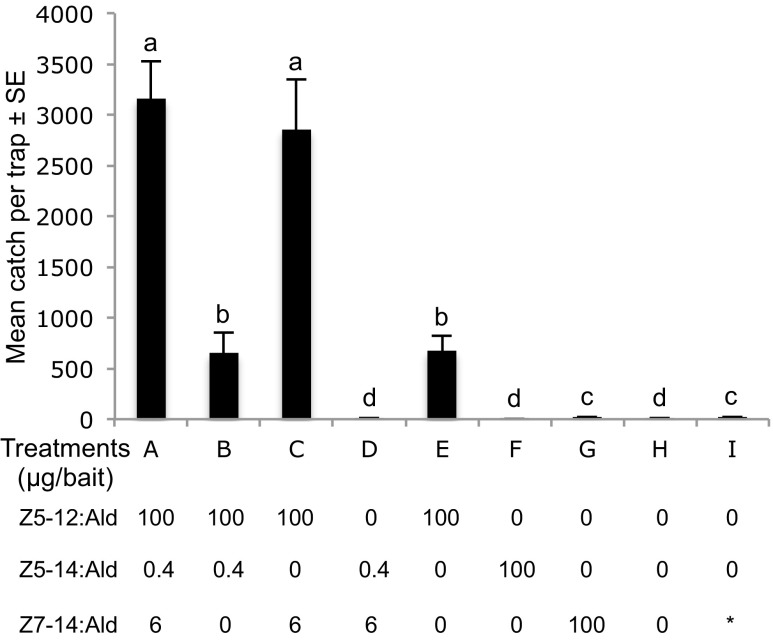
Trap catches of male *Holocacista rivillei* by different pheromone component candidates presented singly and in mixtures in a vineyard located in Avio, Italy (2013). Bars with the same letter are not significantly different (P > 0.05, one-way-ANOVA followed by a LSD test; 5 replicates for each treatment). Asterisk indicates that the treatment I is the commercial bait of the olive moth, *Prays oleae* containing a single component Z7–14:Ald at unknown concentration

The first round of field-trapping experiments in Italian vineyards showed that for *A. oinophylla*, the binary mixture of Z5–14:Ald and Z7–14:Ald in a ratio of 15:100 respectively was the most attractive treatment to conspecific males. Addition of Z5–12:Ald to this mixture did not increase the attractiveness, but removal of any of the two C_14_ aldehydes significantly reduced the number of males caught (Fig. 4). As no pheromone candidates had been identified in volatile collections from *H. rivillei*, blends tested were combinations of aldehydes corresponding to the pheromone precursors identified in gland extracts, with Z5–12:Acyl being the most abundant. The combination of Z5–12:Ald and Z7–14:Ald in a ratio of 100:6 trapped the highest number of conspecific males. Addition of Z5–14:Ald did not increase the trap catch further, whereas removal of either Z5–12:Ald or Z7–14:Ald significantly decreased the number of males attracted (Fig. 5).

**Fig. 6:**
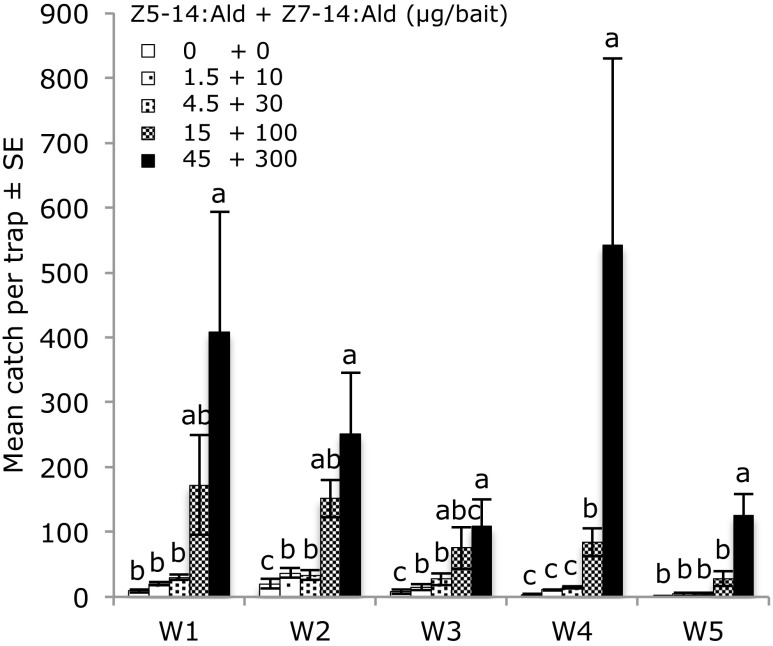
Trap catches of male *Antispila oinophylla* during five weeks (W1-W5, 16th May to 19th June, 2014) by different doses of the binary pheromone blend in the vineyard located in Pochi di Salorno, Italy. Bars with the same letter are not significantly different (P > 0.05, one-way-ANOVA followed by a LSD test; 5 replicates for each treatment)

**Fig. 7:**
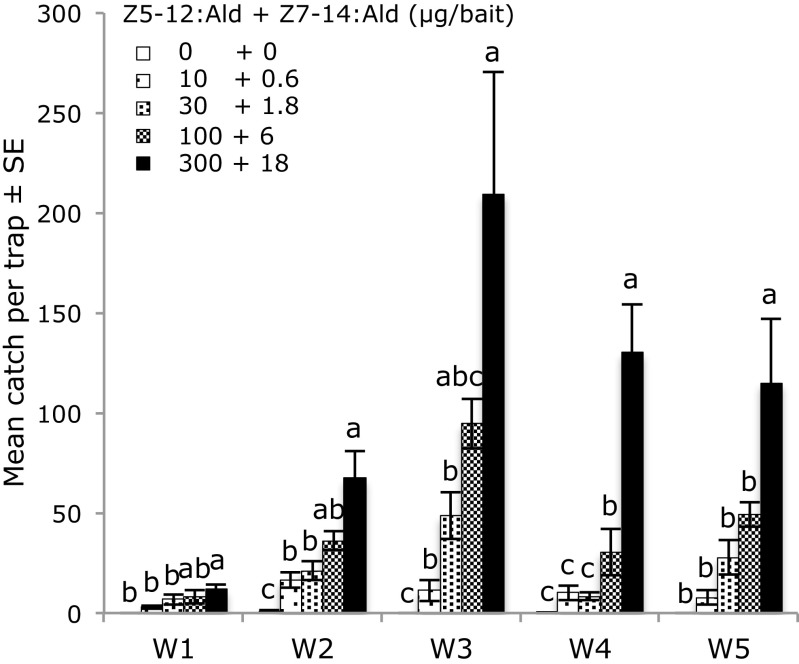
Trap catches of male *Holocacista rivillei* during five weeks (W1-W5, 22nd May to 23rd June, 2014) by different doses of the binary pheromone blend in the vineyard located in Avio, Italy (2014). Bars with the same letter are not significantly different (P > 0.05, one-way-ANOVA followed by a LSD test; 5 replicates for each treatment)

In the second round of field experiments, different doses of the most active blend for each of the species were tested during five weeks. The highest dose of the binary mixture of Z5–14:Ald and Z7–14:Ald (45 + 300 μg/bait) was the most attractive dose to male *A. oinophylla* during all five weeks, although the difference between the highest and the second highest dose (15 + 100 μg/bait) was statistically significant only during the last two weeks (Fig. 6). For *H. rivillei,* the mixture of Z5–12:Ald and Z7–14:Ald was similarly most attractive at the highest dose (300 + 18 μg/bait), although the difference between this dose and the three times lower dose was not statistically significant during the first three weeks (Fig. 7). No significant catches of other species were observed in either of above field experiments.

## Discussion

In this study we identified sex pheromone components of *Antispila oinophylla* van Nieukerken & Wagner and a sex attractant for another grapevine leafminer, *Holocacista rivillei* (Stainton). The combination of Z5–14:Ald and Z7–14:Ald, identified in SPME collections of volatiles from female *A. oinophylla,* proved to be highly effective in trapping conspecific males when presented in a ratio of 15:100. In the case of *H. rivillei* we could not find any pheromone candidates in volatiles collected from female moths. Analysis of pheromone precursors present in the glands of females indicated Z5–12:Ald and Z7–14:Ald were candidate pheromone components and a blend of these two compounds in a ratio of 100:6 respectively attracted large numbers of male *H. rivillei*. In both cases, deletion of either compound in the binary mixtures reduced the trap catch significantly.

Z5–12:Ald, Z5–14:Ald and Z7–14:Ald have only been identified as sex pheromone components in Lepidoptera in a few cases. So far, Z5–14:Ald was only reported as a sex pheromone component in another heliozelid moth, the South African grapevine leafminer, *H. capensis* (Wang et al. 2015), whereas Z7–14:Ald was reported as a pheromone component or sex attractant in the North American noctuid species *Spaelotis clandestina* (Harris) and *Simyra henrici* Grote (now a synonym of *Acronicta insularis* (Herrich-Schäffer)) (Steck et al. 1982a, b), in some species of *Prays* (Praydidae, Yponomeutoidea) (Campion et al. 1979; Nesbitt et al. 1977; Renou et al. 1979; Vang et al. 2011) and in the leafminer *H. capensis* (Wang et al. 2015). In addition Z7–14:Ald was recorded as an attractant for the heliozelid *Antispila treitschkiella* (Fischer von Röslerstamm) (Tóth et al. 1992). The Z5–12:Ald was found as sex pheromone component in *Gastropacha quercifolia* (Linnaeus) (Bestmann et al. 1993) and some noctuid moths in the genus *Euxoa* (Byers et al. 1981; Underhill et al. 1981).

Collection of *A. oinophylla* pheromone samples using SPME proved to be sufficient for both GC/MS and GC-EAD analyses. However, collection from *H. rivillei* females did not produce samples with EAD activity or any compounds appearing as obvious pheromone component candidates in GC/MS analyses. Attempts using an organic solvent (heptane) to extract the terminal abdominal tips were also unsuccessful (data not shown). Fewer individuals of *H. rivillei* were available to us and we tried an alternative approach, performing a comparative analysis of the pheromone gland fatty acyl precursors in the two species. Type I moth pheromones are biosynthetically derived along conserved pathways, including desaturation and limited chain shortening (For a recent review see Löfstedt et al. 2016). The fatty acid precursors of Z5–14:Ald and Z7–14:Ald are oleic acid ((*Z*)-9-octadecenoic acid) and palmitoleic acid ((*Z*)-9-hexadecenoic acid). The ratios of the corresponding 14 carbon fatty acids in the glands of *A. oinophylla* were similar to the ratio between the two aldehydes. The amount of Z5–14:Acyl was very low in *H. rivillei* but instead a large amount of Z5–12:Acyl was found together with Z7–14:Acyl. The 12-carbon homolog is derived from the Z7–14:Acyl by another round of chain-shortening and its abundance suggested that Z5–12:Ald could be a pheromone component in this species. For the field experiment on *H. rivillei*, the blend ratio of the binary mixture of pheromone component candidates was set according to the ratio of corresponding Z5–12 and Z7–14:Acyl precursors, and this mixture turned out to be a potent sex attractant for male *H. rivillei*. Sex attractants are defined as chemicals that attract males (or females) of a given species, but for which conspecific females (or males) have not yet been shown to produce the compounds (Löfstedt et al. 2016).

As with *A. oinophylla*, *H. capensis* from South Africa also uses Z5–14:Ald and Z7–14:Ald as essential sex pheromone components, but in a different blend ratio (Wang et al. 2015). The corresponding precursors, Z5–14:Acyl and Z7–14:Acyl were present in the pheromone gland of female *H. capensis* in the same ratio as the pheromone components, suggesting a metabolic consistency in the relation between the fatty acyl precursors and the final pheromone components in this group of moths.

*Holocacista capensis* is native to South Africa and *A. oinophylla* is a North American species recently established in Italian vineyards, whereas *H. rivillei* is a native European species. During their independent evolutionary history, cross-attraction among these species does not seem to be a possible event due to the geographical barriers. Their divergence in the pheromone components or in the blend ratio might have been driven by presence of other sympatric species. On the receiver side, males of *H. rivillei* and *A. oinophylla* both responded strongly to all the three identified compounds in EAG recordings (Fig. 3), although Z5–12:Ald and Z5–14:Ald are not essential attractants for *A. oinophylla* and *H. rivillei*, respectively. This may be because the pheromone receptors are not specific and respond also to analogues or alternatively receptors tuned to the non-specific compounds are actually present and play a role in interspecific interactions. Mating communication in the Heliozelidae species studied so far seems to depend exclusively on aldehydes. However, screening of EAG-activities of acetates and alcohols corresponding to the aldehyde pheromones could provide additional information on the specificity of the antennal response and the potential role of these compound classes in pheromone communication in the Heliozelidae.

Including this study, sex pheromones and attractants have now been identified in four heliozelid species. These species represent a clade that comprises most global Heliozelidae, including all Vitaceae-feeding heliozelid leafminers (*Antispila* group I, *Holocacista* group and *Antispila ampelopsifoliella* group) (Milla et al. 2017). Our findings suggest that the identified pheromone components were present in the ancestor of this clade since it is unlikely that the same pheromones evolved independently two or three times. We suggest that other heliozelid species use the same or similar compounds as pheromone components, possibly with specific blend ratios to achieve premating reproductive isolation.

In dose response experiments the highest dose tested was consistently the most attractive for both species over five weeks. Thus, it cannot be ruled out that a higher dose would have been even more attractive. Catches fluctuated between weeks. For *A. oinophylla* the highest catch was recorded during week four and for *H. rivillei* the highest catches occurred during the last three weeks. It is likely that fluctuating trap catches reflected differences in moth abundance over the flight period and the results also confirmed that the baits maintain their attractiveness for at least five weeks. The pheromone lures reported in this study can be used in monitoring of these vineyard pests and potentially also for pheromone-based population control by mating disruption.
